# Glycosylated Metal Phthalocyanines †

**DOI:** 10.3390/molecules201119683

**Published:** 2015-11-10

**Authors:** Michael Hanack

**Affiliations:** Institut für Organische Chemie der, Universität Tübingen, Auf der Morgenstelle 18, 72076 Tübingen, Germany; hanack@uni-tuebingen.de; Tel.: +49-7071-297-2432; Fax: +49-7071-295-268

**Keywords:** phthalonitriles, phthalocyanines, glycosylation

## Abstract

In the first part; the syntheses of mono-; di-; and tetra-glycosylated phthalonitriles is described; which are the most used starting materials for the preparation of the corresponding glycosylated metal (mostly zinc) phthalocyanines. In the second section; the preparation of symmetric and unsymmetric mono-; tetra-; and octa- glycosylated zinc phthalocyanines are reviewed; in which the sugar is attached to the phthalocyanine macrocycle; either anomerically or via another one of its OH-groups.

## 1. Introduction

**Figure 1:**
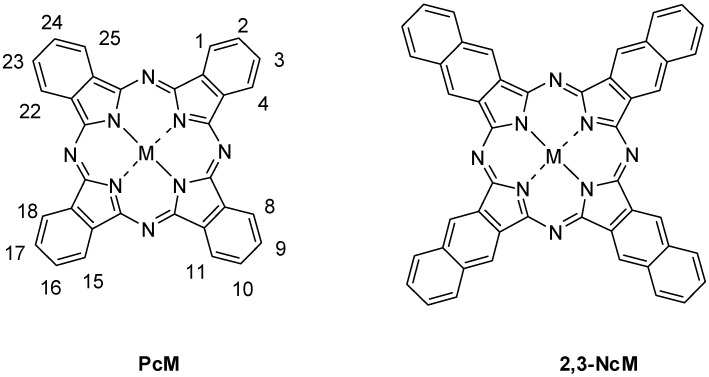
Structures of PcM and 2,3-NcM.

Phthalocyanines (Pcs), (Figure 1), PcM metal phthalocyanines and naphthalocyanines (Ncs), respectively, are macrocyclic compounds which are structurally related with porphyrins and porphyrazines. Phthalocyanines, although not found in nature, have resemblance with naturally-occurring substances, such as hemoglobin, vitamin B_12_, and chlorophyll. Phthalocyanines have found many applications in various fields [1,2,3,4,5].

**Figure 2:**
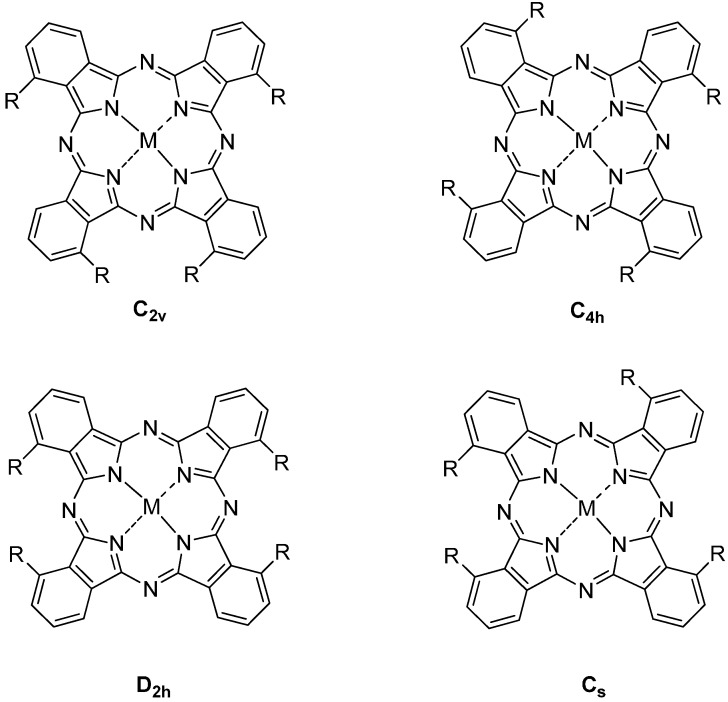
Constitutional isomers of tetrasubstituted metal phthalocyanines.

Phthalonitriles and substituted phthalonitriles are the most widely used starting materials for the synthesis of metal free and metal phthalocyanines. Substituted Pcs are generally synthesized from the corresponding substituted phthalonitriles. As an example, tetrasubstituted Pcs are obtained as a mixture of four constitutional isomers (C_2v_, C_4h_, *etc.*) (Figure 2) by tetramerization of e.g., 3-substituted phthalonitriles.

Separation of the isomers of substituted Pc was first achieved by Hanack and coworkers using high-performance liquid chromatography (HPLC) [3,5].

The solubility of phthalocyanines in common organic solvents is low but can be increased by introduction of bulky or long chain substituents in the periphery of the macrocycle and/or by axial ligand substitution of an appropriate central metal e.g., Si, Ge, or with central metals like Fe or Ru with coordinating axial ligands (axial substitution) [2,3,4,5,6,7,8,9,10,11]. For a discussion of the spectral properties of Pcs see ref. [2,4]. Phthalocyanines have first found use as dyes and pigments, however, more recently they have been employed in a variety of high-tech fields, e.g., as semiconductors, in photovoltaic solar cells, electrophotography, rectifying devices, molecular electronics, Langmuir-Blodgett films, liquid crystals, very intensively in nonlinear optics (optical limiting), photodynamic reagents for cancer therapy (PDT), and other medical applications [9,10,12,13,14,15].

In 1985 Ben-Hur *et al.* [12] showed that some Pcs can photosensitize inactivation of mammalian cells. The ability of Pcs to act as second generation photosensitizers is due to the long wavelength band around 600–750 nm with large extinction coefficients. The presence of diamagnetic metals e.g., Zn, Al, Ga *etc.* in the Pc core enhance the photo activity due to a long-lived triplet state, leading to the generation of higher concentration of singlet oxygen (^1^O_2_), with quantum yields of 0.18–0.62 [16,17,18,19]. However, the low solubility and high aggregation tendency of phthalocyanines in aqueous medium leads to a decrease in the excited singlet states by internal conversion [16,17,18,19]. Our group has worked for many years on the synthesis and applications of metal phthalocyanines.

The interest for glycosylated metal phthalocyanines was stimulated by comparing the extensive work on glycosylated porphyrins with the almost unknown phthalocyanine counterparts and their application in PDT. It was assumed that the presence of carbohydrate substituents in Pcs would increase their solubility in aqueous media, their membrane activity and, thereby, increase their tumor selectivity when applied in PDT.

Carbohydrate substituted pthalocyanines were not known until 1989, when Maillard *et al.* published the first paper on a glucofuranose substituted PcZn [20]. For details see page 5. Since 2006 when Hanack, Ziegler *et al.* [21] prepared the first anomerically-substituted zinc phthalocyanines, additional glycosylated metal Pcs and Ncs were synthesized by our and other groups.

The most common starting materials for the syntheses of glycosylated phthalocyanines are glycosylated phthalonitriles.

## 2. Glycosylated Phthalonitriles

**Scheme 1:**
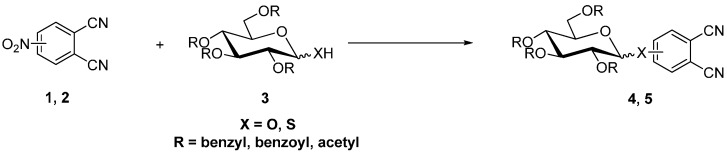
Glycosylation reaction between 3- or 4-nitrophthalonitriles (**1**, **2**) and glycopyranoses (**3**).

A well-known reaction for the preparation of phthalonitriles is the cyano-dehalogenation process (Rosenmund von Braun reaction) [22] in which 1,2-dibromobenzenes are reacted with cuprous cyanide in refluxing DMF. Hanack and co-workers proposed an even easier method to prepare substituted phthalonitriles from substituted catechols via their corresponding aryl bistriflates [23]. They also developed a palladium-catalyzed cyanation of mono- and disubstituted *o*-dibromobenzenes to obtain the corresponding phthalonitriles [24]. A few examples for the synthesis of mono- and polyglycosylated phthalonitriles will be given: by S_NAr_ displacement of NO_2_-groups monoglycosylated phthalonitriles e.g., **4** or **5** with various sugar molecules were synthesized [20,21] by nitrite substitution in 4- or 3-nitrophthalonitriles **1** and **2** with anomerically-deprotected glycosides and thioglucosides in DMF or DMSO, while NaH or K_2_CO_3_ was used as base [25,26,27,28] (Scheme 1).

In glycosylated phthalonitriles **6** and **7** the sugar units are linked to the phthalonitrile fragment by a hydroxyl group located at carbon C-6 of the glycoside [27,28,29,30,31]. **6** and **7** were synthesized by nucleophilic displacement of nitro groups in the 4- or 3-nitrophthalonitriles **1**, **2** with the corresponding sugars.


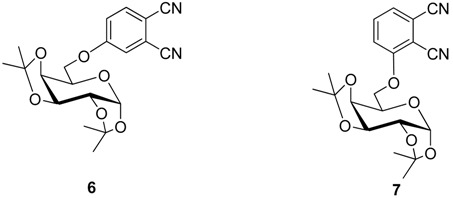


**Scheme 2:**
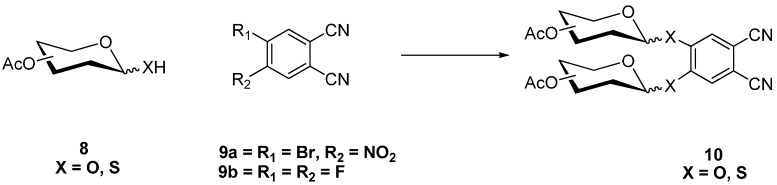
Synthesis of 4,5-diglycosylated phthalonitriles **10**.

4,5-Diglycosylated phthalonitriles e.g., **10** were prepared for the first time by Hanack, Ziegler, and coworkers (Scheme 2) [32] from 4,5-difluorophthalonitrile (**9b**) and anomerically-deprotected sugars, using K_2_CO_3_ as base.

**Scheme 3:**
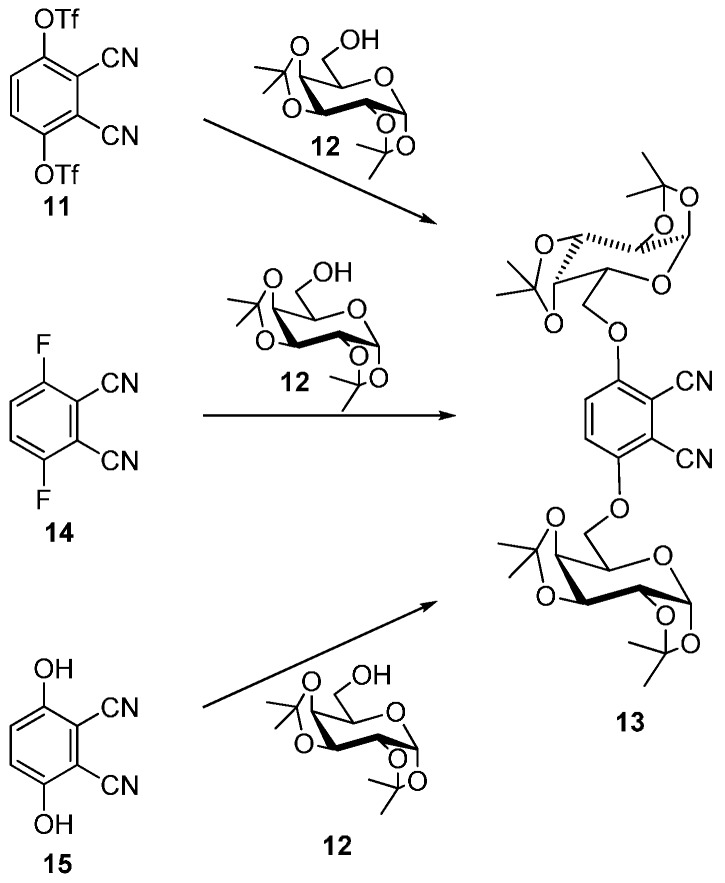
Different methods for the synthesis of 3,6-substituted phthalonitriles.

4,5-Diglycosylated phthalonitriles, in which two galactose units connected via carbon-6, were also synthesized using the same method [30,33,34,35]. Several attempts for the connection of a carbohydrate residue in the positions 3 and 6 of phthalonitrile were published by Hanack, Ziegler *et al.* recently (Scheme 3) [36]. First 3,6-bis (triflyl) phthalonitrile (**11**) was reacted with 1,4-bis(1,2,3,4-di-*O*-isopropylidene-α-d-galactopyranose (**12**) in DMF to give 1,4-bis(1,2:3,4-di-*O*-isopropylidene-*α*-d-galactopyranos-6-yl)-phthalonitrile (**13**) in a yield of 17%. Next **13** was also synthesized by nucleophilic displacement of two fluorine atoms in 3,6-difluorophthalonitrile (**14**) with galactopyranose **12** and NaH in toluene (yield 37%). Finally, 2,3-dicyanohydroquinone (**15**) dissolved in toluene was reacted under Mitsunobu conditions (diethylazodicarboxylate, triphenylphosphine) to give sugar phthalonitrile **13** in 46% yield.

**Scheme 4:**
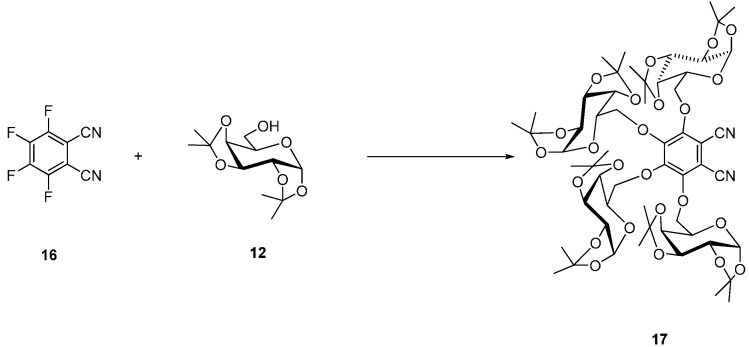
Synthesis of tetrakis (1,2,3,4-di-*O*-isopropylidene-α-d-galactopyranose-6-yl)phthalonitrile (**17**).

Nucleophilic substitution of four fluorine atoms in tetrafluorophthalonitrile (**16**) by 1,2:3,4-di-*O*-isopropylidene-α-d-galactopyranose units (**12**) gave the corresponding tetraglycosylated phthalonitrile **17** (Scheme 4) [33].

Only one example of a glycosylated naphthalonitrile **18** is reported by the group of Hanack and Ziegler [37].


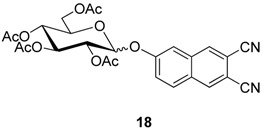


## 3. Glycosylated Phthalocyanines

The carbohydrate substituents can be attached to the Pc (or Nc) macrocycle through a glycosidic (anomeric) bond with a specific anomeric configuration, as a mixture of anomeric configurations or via one of its other functional groups. In addition the carbohydrate substituents can be attached to the macrocycle either in the α- or β-configuration, generating many isomers, which are difficult to separate in pure form.

Tetrasubstituted Pcs form four constitutional isomers (see Figure 2).

The first carbohydrate-substituted zinc(II) phthalocyanine **19** in which the sugar is linked via a non-anomeric bond was reported in 1989 by Maillard *et al.* [20] and later by Ng *et al.* [26].


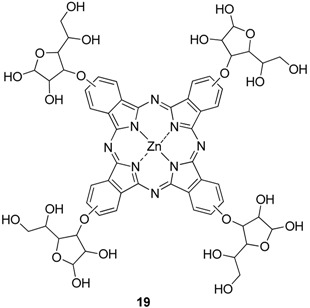


**Scheme 5:**
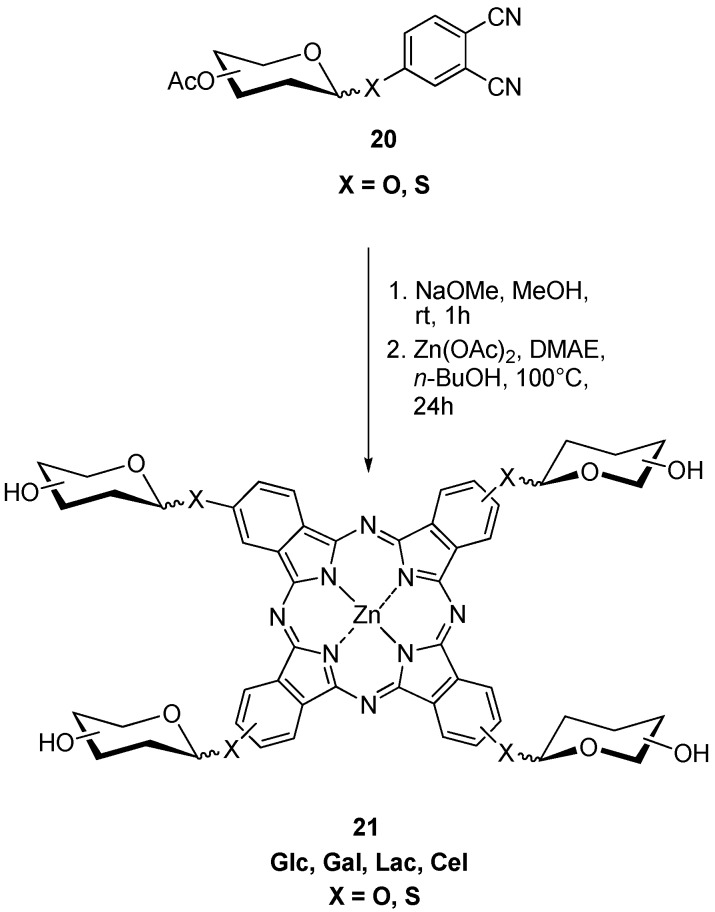
Synthesis of anomerically-glycosylated zinc(II) phthalocyanines **21**.

The first *anomerically*-glucosylated zinc(II) phthalocyanine **21** was synthesized 2006 by Hanack, Ziegler *et al.* as shown in Scheme 5 [21]. Hanack, Ziegler, and co-workers then, in 2006, developed a general method for the synthesis of anomeric tetra carbohydrate substituted PcZns **21** starting with corresponding phthalonitriles **20** in which sugar moieties such as glucose, galactose, maltose, thioglucose, and thiogalactose, are attached at β-position of the Pc macrocycle (Scheme 5) [28].

Additionally, tetraglycosylated PcZns **22** with the substituents at the α-position of the phthalocyanine ring were prepared by the same authors. As sugar substituents glucose, galactose, maltose, cellobiose, and lactose containing O or S atoms at anomeric position were selected [38].


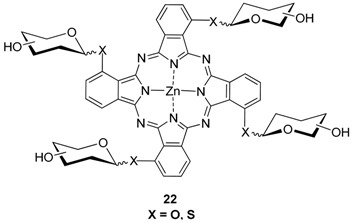


**Scheme 6:**
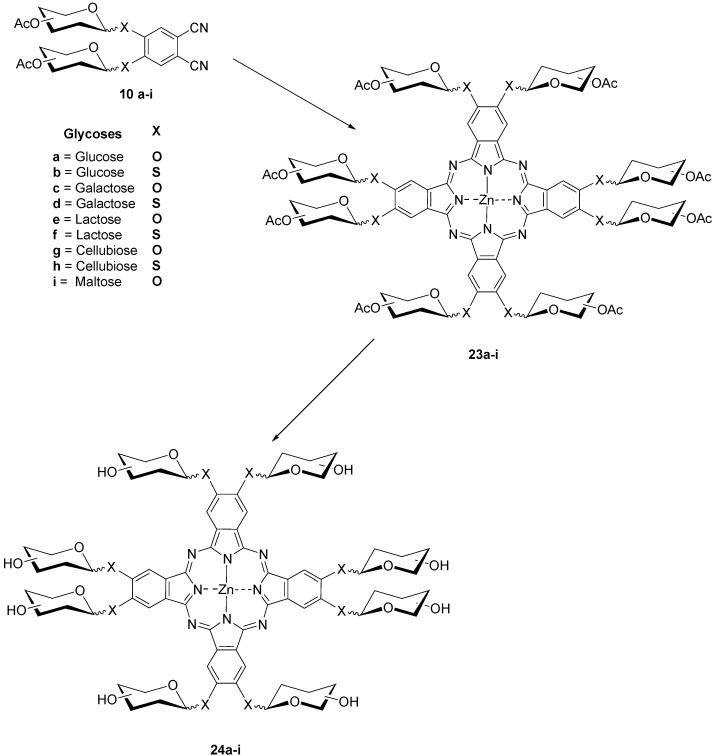
Synthesis of octaglycosylated zinc (II) phthalocyanines **24**.

The syntheses of the anomeric octaglycosylated phthalocyanines **23a**–**i** and **24a**–**i**, respectively, (Scheme 6) turned out to be rather difficult; several methods had to be applied before a successful route could be found [32].

**Scheme 7:**
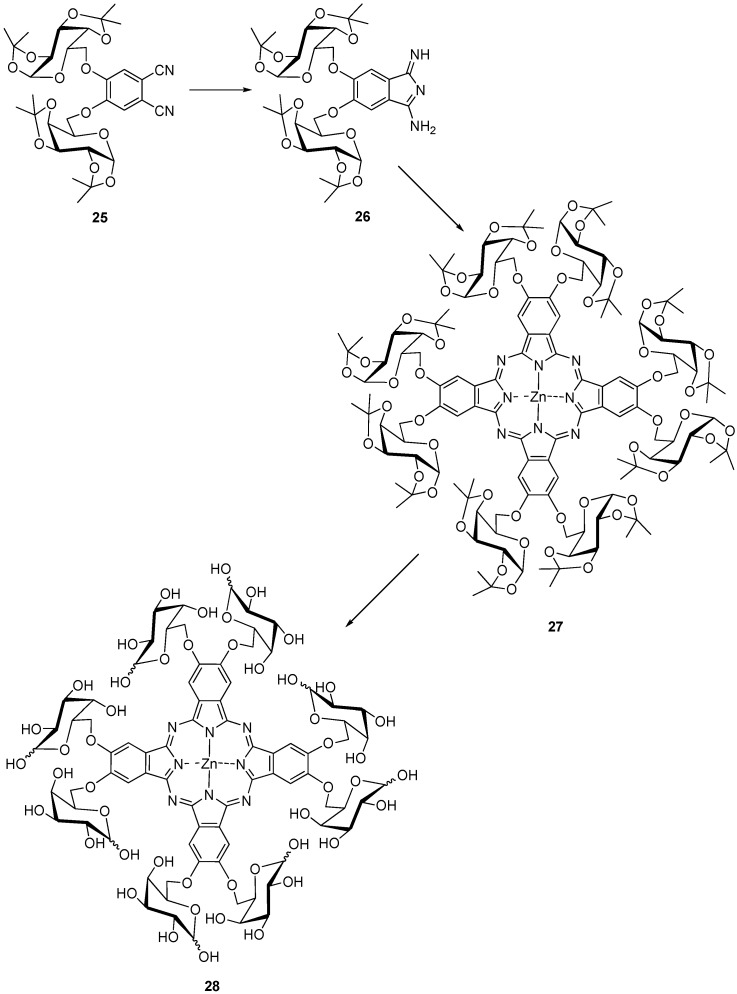
Synthesis of octa-substituted galactose zinc(II) phthalocyanine **28**.

After several attempts using a variety of conditions **10a** was heated in DMF with 30 mol % hexamethyldisilazane (HMDS), *p*-toluenesulfonic acid and Zn(OAc) at 125–130 °C overnight [34,35]. Using this procedure the octaglycosylated phthalocyanines **23a**–**i** were obtained then in yields between 55% and 78% and deprotected to form the PcZns **24a**–**i** [32,34,35]. **24a**–**i** gave high triplet quantum yields ranging from 0.68 to 0.88 [39]. Attempts to cyclize compound **25** under various conditions which had been previously found suitable [21,29,31] failed. Therefore, **25** was first transformed into the isoindoline **26** [34,35] which could be converted into PcZn **27** in 29% yield as a green amorphous solid. Deprotection of the sixteen isopropylidene groups in **27** was achieved by treating it with aqueous trifluoroacetic acid. The obtained PcZn **28** is highly soluble in water (Scheme 7).

A number of other glycoconjugated zinc(II) phthalocyanines were also studied by Torres and Ng, respectively, concerning their effectiveness in PDT [25,26,29,30,31,33].

**Scheme 8:**
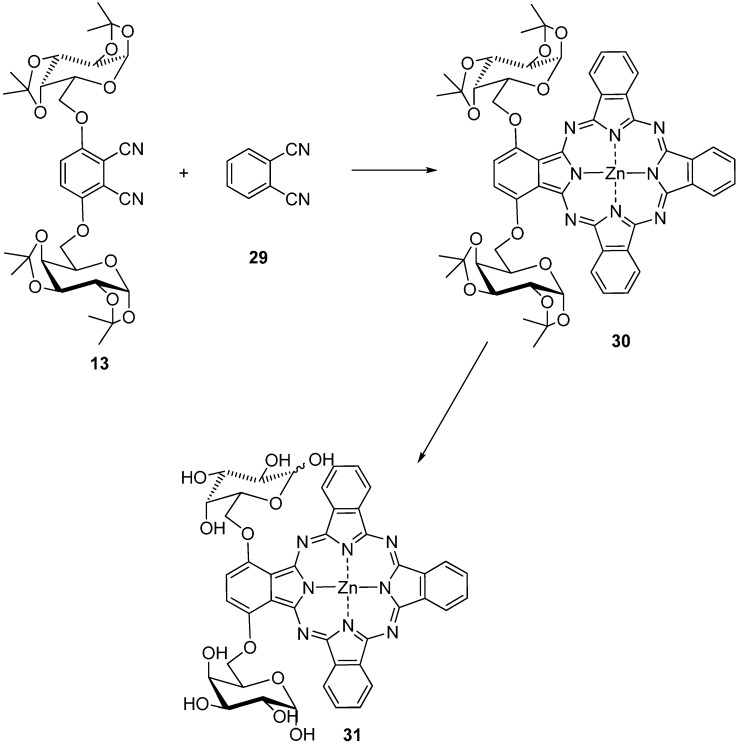
Synthesis of the unsymmetric Pc **31**.

By condensation of 1,4-bis(1,2:3,4-di-*O*-isopropylidene-*α*-d-galactopyranos-6-yl)-phthalonitrile (**13**) with an excess of phthalonitrile (**29**) and zinc bromide 22% of [1,4-bis(1,2:3,4-di-*O*-isopropylidene-α-d-galactopyranos-6-yl)-phthalocyaninato]zinc(II) (**30**) was obtained which was deprotected to form **31** [36] (Scheme 8).

Another water soluble unsymmetrical phthalocyanine **32**. was synthesized by condensation of tetrakis(1,2:3,4-di-*O*-isopropylidene-α-d-galactopyranos-6-yl)phthalonitrile with phthalonitrile and zinc(II) acetate and subsequent deprotection of the isopropylidene groups [33].


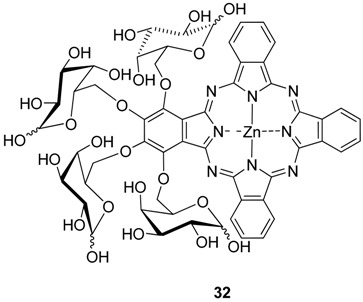


Starting from the corresponding substituted phthalonitriles other water soluble unsymmetrical mono **33**, **34**, and tetra-glycosylated phthalocyanines **35**, **36** with sugar units linked by the hydroxyl group located at carbon C-6 were synthesized [26].


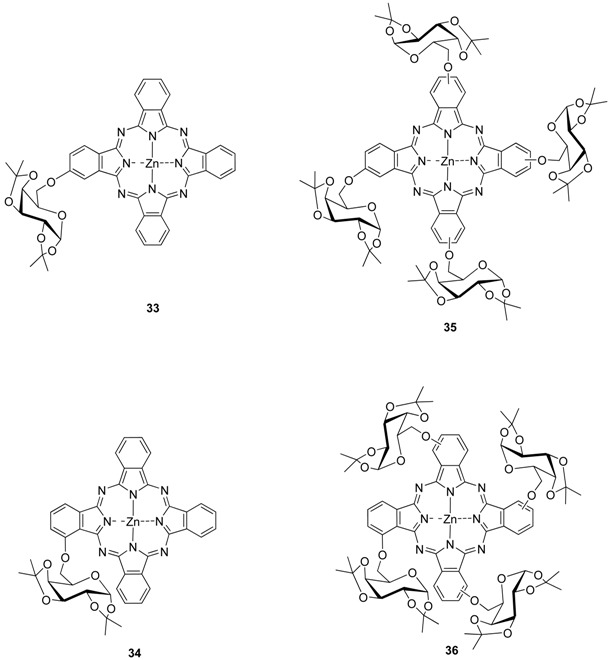


Protected zinc(II) phthalocyanines **33**–**36** were not significantly aggregated in organic solvents, giving a weak to moderate fluorescence emission. Upon irradiation **33**–**36** sensitize the formation of singlet oxygen in DMF, with quantum yields in range of 0.40–0.66. The *in vitro* PDT activities of these zinc(II) phthalocyanines against HepG2 human hepatocarcinoma and HT29 human colon adenocarcinoma cells were also studied. The mono-glycosylated phthalocyanines **33**, **34** show significantly higher photocytotoxicity compared with the tetra-α-glycosylated analogues **35**, **36**, exhibiting IC_50_ values down to 0.9 µM [26].

**Scheme 9:**
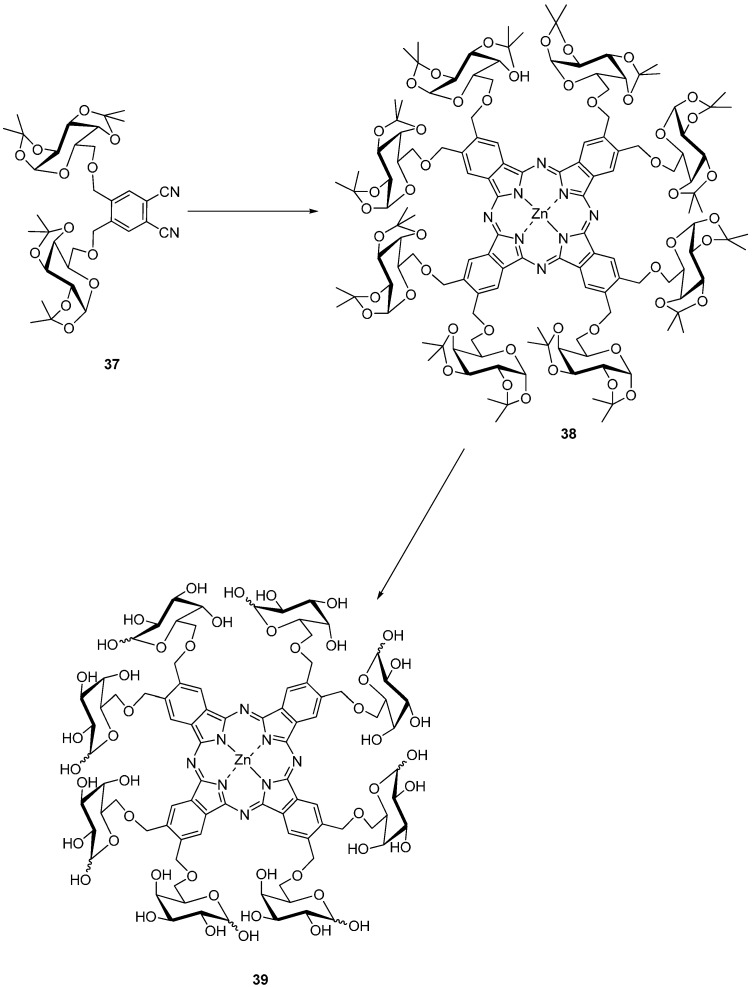
Synthesis of octa-substituted galactose zinc(II) phthalocyanine **39**.

The octa-substituted galactose zinc(II) phthalocyanines **38**, **39** have been reported by Torres *et al.* (Scheme 9) [30].

**Scheme 10:**
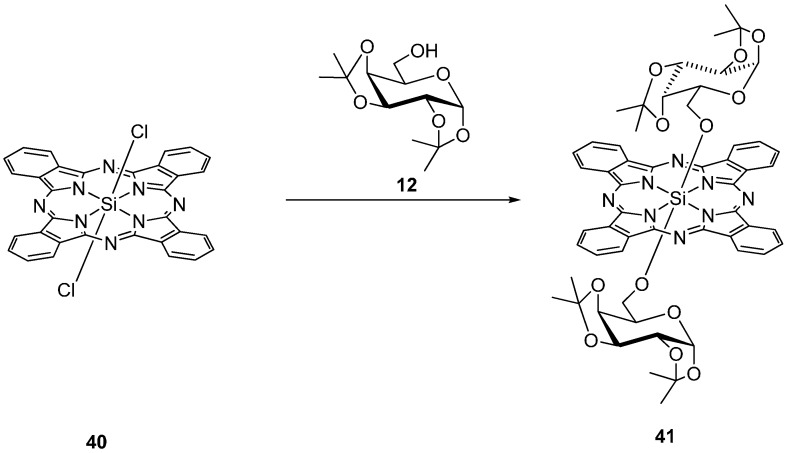
Synthesis of PcSi **41** with axial protected galactose substituents.

A series of silicon(IV) phthalocyanines with one or two axial acetal-protected galactose substituent(s) e.g., **41** have been prepared by Ng *et al.* starting with dichlorosiliconphthalocyanine **40** (Scheme 10) [40,41,42].

The future experimental work in our laboratory will concentrate on the synthesis of water soluble naphthalocyanines.

## 4. Conclusions

This short review deals with the synthesis of glycosylated metal (mostly Zn) phthalocyanines which e.g., are potential reagents for photodynamic cancer therapy (PDT). The main starting materials for glycosylated zinc phthalocyanines are mono-, di-, and tetra glycosylated phthalonitriles of which the sugar part is e.g., glucose, galactose, lactose, cellobiose, maltose, and others. The synthesis of the glycosylated phthalonitriles is described. Then the transformation of these phthalonitriles into symmetrically and unsymmetrically mono-, tetra-, and octa- glycosylated zinc phthalocyanines is outlined. The sugars are attached either anomerically or via another of its OH-groups to the phthalocyanine macrocycle.
